# Supplementary material for: Data-driven prediction of complex crystal structures of dense lithium
Source: Nat Commun. 2023 May 22;14:2924. doi: 10.1038/s41467-023-38650-y (PMC10203143; doi:10.1038/s41467-023-38650-y)
Supplement: Supplementary file 1 — Supplementary Information [file 41467_2023_38650_MOESM1_ESM.pdf]

Supplementary Information for
Data-Driven Prediction of Complex Crystal Structures of Dense Lithium

Xiaoyang Wang,[1,2,*] Zhenyu Wang,[1,*] Pengyue Gao,[1] Chengqian Zhang,[3,4]
Jian Lv,[1,†] Han Wang,[2,5,†] Haifeng Liu,[2] Yanchao Wang,[1] and Yanming Ma[1,†]

[1]*Key Laboratory of Material Simulation Methods & Software of Ministry of Education and State Key Laboratory of Superhard Materials, College of Physics, Jilin University, Changchun 130012, P.R. China*
[2]*Laboratory of Computational Physics, Institute of Applied Physics and Computational Mathematics, Fenghao East Road 2, Beijing 100094, P.R. China*
[3]*DP Technology, Beijing 100080, P.R. China*
[4]*College of Engineering, Peking University, Beijing 100871, P.R. China*
[5]*HEDPS, CAPT, College of Engineering, Peking University, Beijing 100871, P.R. China*

(Dated: May 18, 2023)

S I. DENSITY FUNCTIONAL THEORY CALCULATIONS

The density functional theory (DFT) calculations were carried out for the construction of training dataset of Deep Potential (DP) models, refinement of predicted structures and enthalpy calculations by employing the VASP code [1], adopting the projector-augmented wave (PAW) method [2], with the generalized gradient approximation proposed by Perdew, Burke and Ernzerhof (PBE) [3, 4]. The $2s^22p^1$ electrons are treated as valence electrons. The electronic wave functions were expanded in a plane-wave basis set with a kinetic energy cutoff (ENCUT) of 1300 eV, and the Monkhorst-Pack k-point sampling with grid spacing (KSPACING) of 0.1 Å^{-1} was chosen to ensure the convergence of total energy, forces, and virial tensors below 1 meV/atom, 10 meV/Å and 1 meV/atom, respectively. Convergence tests on total energy for the cI16 and $C2/m$ structures are shown in Supplementary Fig. **S1**.

Calculations for phonon and Gibbs free energy under harmonic approximation were carried out using the finite displacement method as implemented in the PHONOPY code [5]. The atomic displacement is 0.01 Å. The Hellmann-Feynman forces were calculated using VASP code[1]. The Monkhorst-Pack k-point sampling with a grid spacing of 0.2 Å^{-1} was chosen, the energy cutoff for the plane-wave basis set was set to 600 eV, and the other settings and parameters are consistent with those used in the construction of the DP datasets. Supercells containing 128, 88, 384, and 360 atoms were used for cI16, oC88, oP48, and tI20, respectively. The calculated phonon dispersion curves are shown in Supplementary Fig. **S2**.

The Gibbs free energy under harmonic approximation was also calculated using the density functional perturbation theory as implemented in the ABINIT code [6], with Hartwigsen-Goedeker-Hutter PBE pseudopotential [7] and 250 Ry cutoff. The $15 \times 15 \times 15$ and $4 \times 4 \times 4$ k-point grids were used for the cI16, and oC88, respectively. Dynamical matrices were computed on $5 \times 5 \times 5$ and $4 \times 4 \times 4$ q-point meshes for the cI16 and oC88, respectively.

* These authors contributed equally to the work: Xiaoyang Wang and Zhenyu Wang.
† Corresponding Author: Jian Lv, lvjian@jlu.edu.cn; Han Wang, wang_han@iapcm.ac.cn; Yanming Ma, mym@jlu.edu.cn

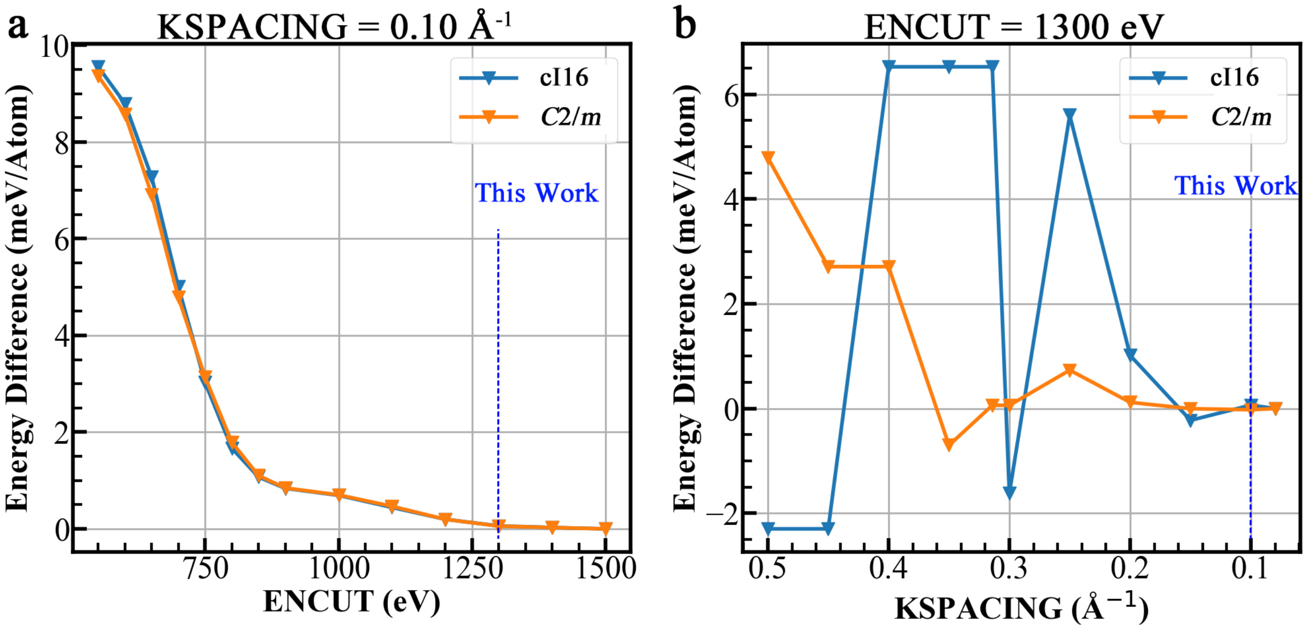

Supplementary Fig. S1. **Convergence tests on VASP calculations.** Convergence tests on total energy in VASP calculations for the cI16 and $C2/m$ structures. Total energies as a function of **a** kinetic energy cutoff (ENCUT) and **b** grid spacing of k-point sampling (KSPACING), relative to the results obtained using more stringent parameters (ENCUT = 1500 eV and KSPACING = 0.08 Å^{-1}).

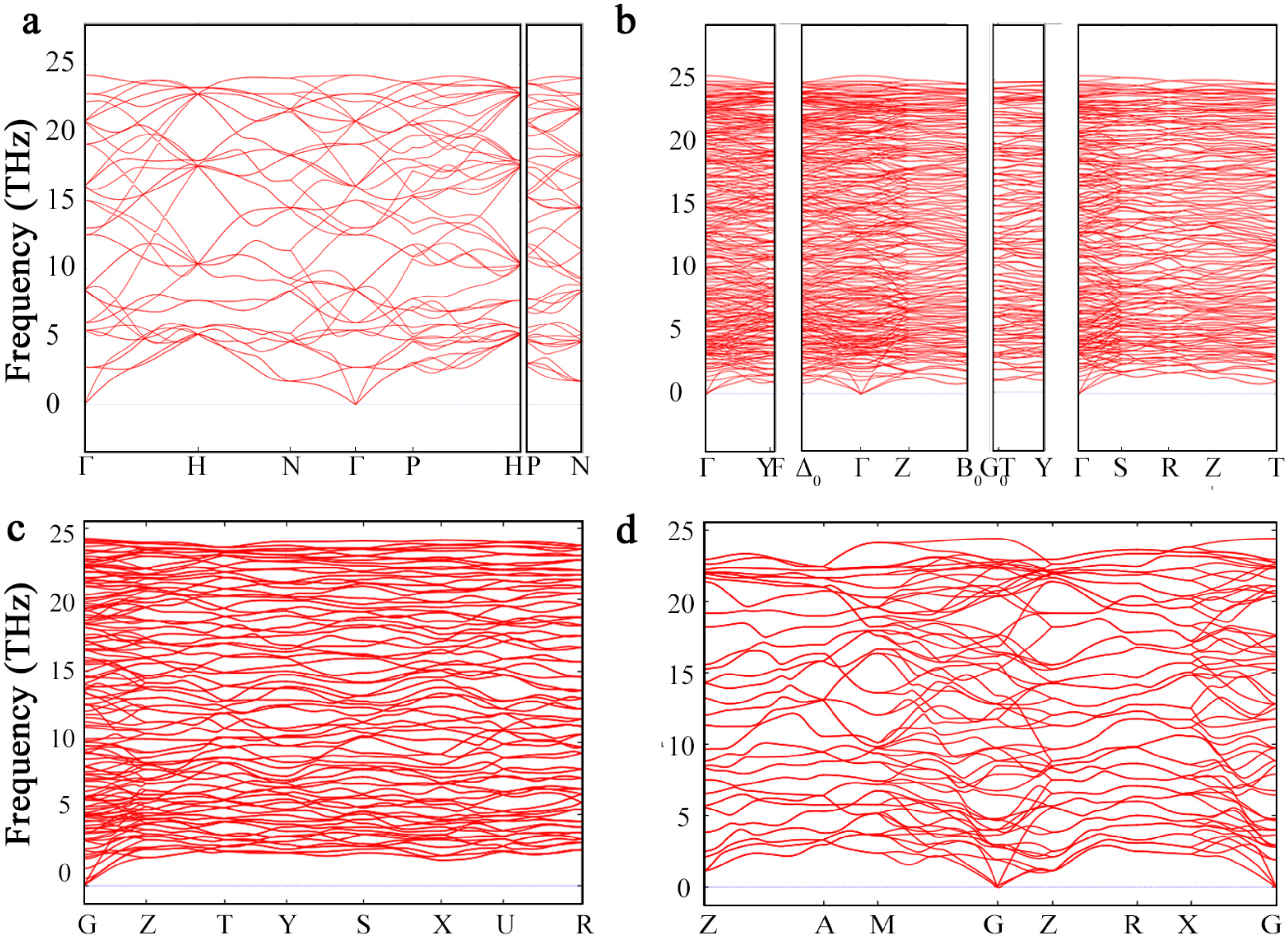

Supplementary Fig. S2. **Phonon dispersion curves of Li phases.** Phonon dispersion curve calculated using the PHONOPY code for **a** cI16 at 65 GPa, **b** oC88 at 65 GPa, **c** oP48 at 60 GPa, and **d** tI20 at 60 GPa.

S II. GENERATION OF THE DEEP POTENTIAL MODELS

The DP models were trained using a highly accurate DFT dataset constructed by the concurrent learning scheme named the Deep-Potential Generator (DP-GEN) [8]. Two DP models were trained. The first model (Li-DP-Hyb1) was initialized from the known experimental phases of Li, and used for accelerating the structure searches. After structure searches, the predicted structures were used to inform the dataset construction of the second model (Li-DP-Hyb2). The Li-DP-Hyb2 model, which contains all information of Li-DP-Hyb1, was used to calculate the Gibbs free energy by thermodynamic integration (TI) through Deep Potential molecular dynamics (DPMD).

A. Protocol for the construction of the dataset

DP-GEN [8] is an efficient tool to construct the most compact and adequate dataset for a DP model. Beginning with a *preliminary dataset*, DP-GEN runs iteratively through the *training*, *exploration* and *labeling* processes.

Preliminary Dataset To initiate the DP-GEN run and guarantee stable sampling in DP-GEN iterations, four initial DP models were firstly trained based on a preliminary dataset, which contains two kinds of configurations:

1. Locally optimized configurations. The preliminary dataset of Li-DP-Hyb1 includes optimized experimental structures: *bcc*, *fcc*, $9R$, cI16, oC88, and oC40 at different pressures. The pressure ranges considered for *bcc*, *fcc*, $9R$, cI16, oC88 and oC40 are [0,40], [0,40], [0,40], [0,75], [40,75], and [40,75] GPa, respectively, with an interval of 5 GPa. The preliminary dataset of Li-DP-Hyb2 additionally includes the predicted structures: aP160, $C2/m$, oP192, oP48, and tI20 from 40 to 75 GPa.

2. Locally perturbed configurations. The locally optimized configurations were then subject to perturbations, with the mean perturbation distance of atoms set to 0.03 Å, and the mean perturbation fraction of cell vectors set to 1.0%. For each structure at each pressure point, 10 perturbations were performed, and included in the preliminary dataset.

Totally, 649 and 1089 configurations were included in the preliminary datasets of the Li-DP-Hyb1 and Li-DP-Hyb2, respectively. The labels (i.e. energies, forces, and virial tensors) of all configurations in the preliminary datasets were obtained by high-accuracy single-point DFT calculations.

Training In each DP-GEN iteration, the first process is the training of four models, using the same training dataset and hyper-parameters, but different random seeds to initialize the model parameters. To enhance the representability of the DP model, we utilized the three-body embedding descriptor and *hybridizes* it with the original smooth edition DP descriptor. The technical details of these descriptors can be found elsewhere[9]. The sizes of the 3 hidden layers of the two-body embedding net, three-body embedding net, and the fitting net are (20, 40, 80), (4, 8, 16) and (240, 240, 240), respectively. The Adam stochastic gradient descent method[10] with default hyper-parameter settings provided by the `TensorFlow` package [11] was used to train the DP models. During the training, the loss function was:

$$\mathcal{L} = \frac{1}{|\mathcal{B}|}\sum_{k\in\mathcal{B}}\Big(p_\epsilon\frac{1}{N}|\hat{E}^k - E^k|^2 + p_f\frac{1}{3N}\sum_{i\alpha}|\hat{F}^k_{i\alpha} - F^k_{i\alpha}|^2 + p_\xi\frac{1}{9N}\sum_{i\alpha}|\hat{\Xi}^k_{\alpha\beta} - \Xi^k_{\alpha\beta}|^2\Big), \tag{1}$$

which measures the accuracy of the model according to the differences between the DFT calculated energy $\hat{E}^k$, forces $\hat{F}^k_{i\alpha}$ and the virial tensor $\hat{\Xi}^k_{\alpha\beta}$, and those predicted by the DP model.

In Eq. (1), $\mathcal{B}$ denotes a mini-batch of datasets, and $|\mathcal{B}|$ is the batch size. The superscript k denotes the index of the training data in the mini-batch. Each training datum contains a configuration (including the coordinates of atoms, the box basis vectors, and the element types) and its corresponding labels. Prefactors (p_ϵ, p_f, p_ξ) are a set of hyper-parameters determining the relative importance of the energy, forces, and virial tensor during the training. The prefactors are gradually adjusted according to the learning rate $r_l(t)$, which exponentially decays with the training step t:

$$r_l(t) = r_l^0 k_d^{\frac{t}{t_d}}, \tag{2}$$

where r_l^0 is the learning rate at the beginning of the training, t_d denotes the typical timescale of the learning rate decaying and k_d denotes the decay rate. The prefactors vary with the learning rate in the following way:

$$p_\alpha(t) = p_\alpha^{limit}[1 - \frac{r_l(t)}{r_l^0}] + p_\alpha^{start}[\frac{r_l(t)}{r_l^0}], \quad \alpha = \epsilon,\ f,\ \text{or}\ \xi, \tag{3}$$

where $p_\alpha(t)$ is either of the three pre-factors (p_ϵ, p_f, p_ξ) at training step t. p_α^{start} and p_α^{limit} are the pre-factors at the beginning and at an infinitely small learning rate, respectively. In practice, a relatively larger force prefactor at the beginning and relatively balanced prefactors at the end of the training can make the best use of the training datasets and achieve relatively good accuracy[12]. Thus, during the DP-GEN iterations, we set $p_\epsilon^{start} = 0.02$, $p_\epsilon^{limit} = 1.00$, $p_f^{start} = 1,000.00$, $p_f^{limit} = 1.00$, $p_\xi^{start} = 0.00$, $p_\xi^{limit} = 0.00$, $t_d = 5000$, $k_d = 0.95$, and $r_0^l = 1 \times 10^{-3}$. The total number of training steps is $1,000,000$.

Exploration In each iteration, the second process is the exploration of configuration space using molecular dynamics. The `LAMMPS` package[13] compiled with the `DeePMD-kit`[14] support was employed to perform the isothermic-isobaric (NPT) DPMD[15] simulations for the exploration. The simulations run up to 5 ps. The timestep during the exploration was set to 1 fs. The pressure and temperature in the DPMD simulations are listed in Supplementary Table **S1**. For the aP160 and oP192 with large unit cells, 64-atom sub-cells are used as the initial configurations of the DPMD simulations. The number of atoms of other structures during exploration is listed in Supplementary Table **S1**.

During the DPMD simulation, we evaluated the error of forces by the deviation of force predictions among the four DP models obtained in the *training* process. If the maximal deviation of atomic forces on a frame in the MD trajectory lies in between an upper bound and a lower bound, the frame is considered as a candidate configuration. At most 150 candidate configurations were labeled by DFT calculations in each DP-GEN iteration. The upper and lower bound were adjusted with respect to the pressure and are listed in Supplementary Table **S1**. As the number of iterations increases, the deviation of force predictions by independently trained DP models is gradually reduced. The convergence of DP-GEN is reached when the deviations of force predictions for 99.9% of explored configurations are below the lower bound.

Supplementary Table S1. The number of atoms, pressure, temperature, lower and upper bound of deviations of force predictions, and whether included in the dataset of Li-DP-Hyb1 and Li-DP-Hyb2 during the *exploration* process in the DP-GEN run.

Structure	Number of Atoms	Sampling Pressure	Temperature	Lower Bound (eV/Å)	Upper Bound (eV/Å)	In Li-DP-Hyb1	In Li-DP-Hyb2
bcc	54	0-40 GPa	10-700 K	0.020	0.20	Y	Y
fcc	64	0-40 GPa	10-700 K	0.020	0.20	Y	Y
cI16	64	5-40 GPa	10-700 K	0.020	0.20	Y	Y
		40-60 GPa	10-300 K	0.023	0.20		
		60-75 GPa	10-400 K	0.025	0.25		
aP160	64	40-55 GPa	10-300 K	0.023	0.20	N	Y
$C2/m$	64	40-55 GPa	10-300 K	0.023	0.20	N	Y
oP192	64	50-60 GPa	10-300 K	0.023	0.20	N	Y
oP48	48	50-75 GPa	10-300 K	0.025	0.25	N	Y
oC88	44	55-75 GPa	10-300 K	0.025	0.25	Y	Y
oC40	40	55-75 GPa	10-300 K	0.025	0.25	Y	Y
tI20	40	55-75 GPa	10-300 K	0.025	0.25	N	Y

Labeling The labels of the candidate configurations, i.e. the energy, force, and virial tensor, were calculated by DFT using VASP[1] code as described in Sec. SI. The candidate configurations and their labels were then appended to the dataset for the model training in the next iteration.

B. Training and refining of the productive model

After the DP-GEN iterations converged, the productive models were trained with longer training steps with the training dataset generated by DP-GEN. To ensure a high training accuracy, the productive models were firstly trained by 9.60×10^7 steps with $p_e^{start} = 0.02$, $p_e^{limit} = 1.00$, $p_f^{start} = 1000.00$, $p_f^{limit} = 1.00$, $p_\xi^{start} = 0.02$, $p_\xi^{limit} = 1.00$, $t_d = 4.80 \times 10^5$, and $r_0^l = 1.00 \times 10^{-3}$. Then, the productive models were additionally trained by 4.80×10^7 steps with $p_e^{start} = 10.00$, $p_e^{limit} = 10.00$, $p_f^{start} = 1.00$, $p_f^{limit} = 1.00$, $p_\xi^{start} = 0.02$, $p_\xi^{limit} = 0.50$, $t_d = 2.40 \times 10^5$, and $r_0^l = 1.00 \times 10^{-4}$. During the training of the productive models, the weight of the locally optimized configurations in the preliminary dataset was increased by ten times.

To further improve the quality of the model, Li-DP-Hyb2 was then subject to refinement training. Refining datasets were constructed for this purpose. Each refining dataset includes two kinds of configurations:

1. Inaccurate ground-state configurations. The quality of the productive model at zero temperature was accessed by reproducing the enthalpy curves of various Li structures. If the error of DP-predicted enthalpy of a structure at a given pressure is larger than 4 meV/atom, or 2 meV/atom near the enthalpy curve minimum in Supplementary

Fig. **S3**, the prediction is considered inaccurate, and the labels of the corresponding configurations are calculated by DFT and appended to the refining dataset.

2. Inaccurate MD configurations. The quality of the productive model was also accessed by labeled DPMD trajectories. The trajectories are obtained by DPMD simulations in NPT ensemble using the experimental and predicted structures as the initial configurations. All structures were simulated at 50 K, 100 K, 150 K, 200 K, and 250 K under different pressure conditions. The *bcc* structure was simulated at 0 GPa and 5 GPa; the fcc structure was simulated at 10 GPa, 20 GPa, and 30 GPa; the cI16 structure was simulated at 45 GPa, 60 GPa, and 70 GPa; the aP160 structure was simulated at 45 GPa; the oP192 structure was simulated at 55 GPa; the oP48 structure was simulated at 60 GPa; the oC88, oC40 and tI20 structures were simulated at 70 GPa. In each thermodynamic condition, DPMD runs up to 5 ps with 1 fs timestep. A frame was labeled every 0.5 ps. Totally, there are 700 labeled frames in all the trajectories. If the testing error of energy at any trajectories is larger than 2.0, 4.0, 6.0, 8.0, and 10.0 meV/atom, at 50, 100, 150, 200, and 250 K, respectively, we considered the prediction inaccurate. The labels of all frames in all DPMD trajectories were then appended to the refining dataset.

Before each refinement training, the refining dataset was merged with the training dataset. The hyper-parameters during the refinement training were $r_0^l = 1.00 \times 10^{-4}$, $p_e^{start} = 10.00$, $p_e^{limit} = 10.00$, $p_f^{start} = 1.00$, $p_f^{limit} = 1.00$, $p_\xi^{start} = 0.02$, $p_\xi^{limit} = 0.50$, $t_d = 8.00 \times 10^4$, and the total number of steps for each refinement training is set to 1.60×10^7. Li-DP-Hyb2 was refined repeatedly until none of the predictions on the enthalpies and MD trajectories were considered inaccurate. Totally, four refining datasets, which consist of nearly 3,000 labeled configurations, were merged into the training dataset of Li-DP-Hyb2 during the refinements. There is no refinement training for Li-DP-Hyb1.

C. Reliability of the Li-DP-Hyb2

The reliability of Li-DP-Hyb2 was accessed by two validations: the prediction on enthalpy curves, and testing on an independent dataset obtained from DPMD with wide thermodynamic conditions.

Prediction of the enthalpy curves. The enthalpies relative to cI16 of various Li structures as a function of pressure are shown in Supplementary Fig. **S3**. The Li-DP-Hyb2 model can accurately reproduce the enthalpy curves for various Li structures. Especially, at the pressure around the minimum of an enthalpy curve, the errors are well below 1.0 meV/atom. Moreover, the Li-DP-Hyb2 can predict the nearly degenerated enthalpies of oC88 and tI20.

Accuracy on an independent testing dataset. The reliability of the Li-DP-Hyb2 model was also validated by the prediction on an independent testing dataset. The independent testing dataset was constructed in a similar way as in the construction of the refining dataset, but obtained from longer DPMD trajectories (10 ps, 50 frames on each trajectory), and covered a wider range of thermodynamic conditions, as listed in Supplementary Table **S2**. Totally, 7,000 frames were included in the independent testing dataset. The root mean square error (RMSE) of the predicted energy of each phase on their sampling thermodynamic condition is listed in Supplementary Table **S2**. The RMSE below 50, 100, 150, 200, and 250 K are 0.68, 0.73, 1.02, 1.21, and 1.39 meV/atom, respectively. The comparisons between the DP-predicted and the DFT-calculated energies of various configurations from 45 to 70 GPa in the independent testing dataset are shown in Supplementary Fig. **S4**.

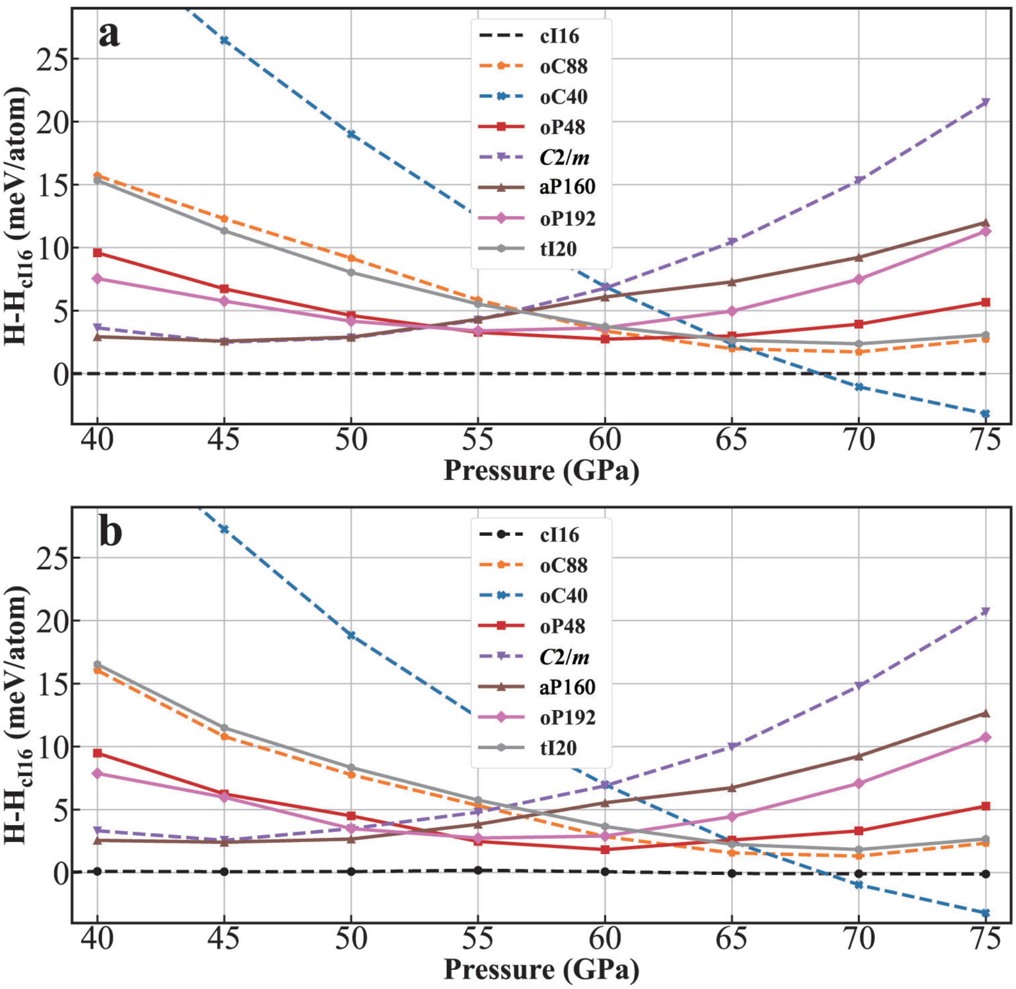

Supplementary Fig. S3. **Relative enthalpies of Li phases.** Relative enthalpies per atom as a function of pressure between 40 and 75 GPa calculated by **a** VASP and **b** Li-DP-Hyb2 model. All enthalpies are relative to the VASP-calculated enthalpies of the cI16 structure. Dashed and solid lines indicate the experimental and predicted structures, respectively.

Supplementary Table S2. RMSEs of energy predicted by Li-DP-Hyb2 on the independent testing dataset for all structures at different thermodynamic conditions. (Units: meV/atom)

Structure	Pressure (GPa)	T = 50 K	T = 100 K	T = 150 K	T = 200 K	T = 250 K
9R	0	0.141	0.315	0.220	0.303	0.334
bcc	0	0.191	0.480	0.499	0.763	1.064
	5	0.295	0.462	0.632	1.299	1.278
fcc	10	0.117	0.187	0.294	0.306	0.389
	20	0.099	0.363	0.393	0.464	0.701
	30	0.314	0.412	0.468	0.760	1.054
aP160	45	0.308	0.745	1.122	1.266	1.412
oP192	55	1.118	1.217	4.706	2.181	4.850
cI16	45	0.171	0.331	0.406	0.511	0.680
	60	0.332	0.351	0.518	0.973	1.245
	70	0.251	0.313	0.464	0.631	1.035
oC88	55	1.093	1.521	1.403	3.311	3.197
	60	0.784	1.461	1.612	2.978	3.831
	65	0.462	1.103	1.472	2.258	3.068
	70	0.426	0.815	1.393	1.798	2.943
oP48	55	0.375	0.950	1.531	1.679	1.998
	60	0.535	0.990	1.192	1.488	1.860
	65	0.650	1.043	0.975	1.155	1.559
	70	1.075	1.273	1.305	1.570	2.000
tI20	55	0.783	1.586	2.640	3.874	4.852
	60	0.707	1.299	2.469	3.515	6.700
	65	0.679	1.302	1.754	3.090	3.277
	70	0.576	0.977	1.503	1.391	1.931

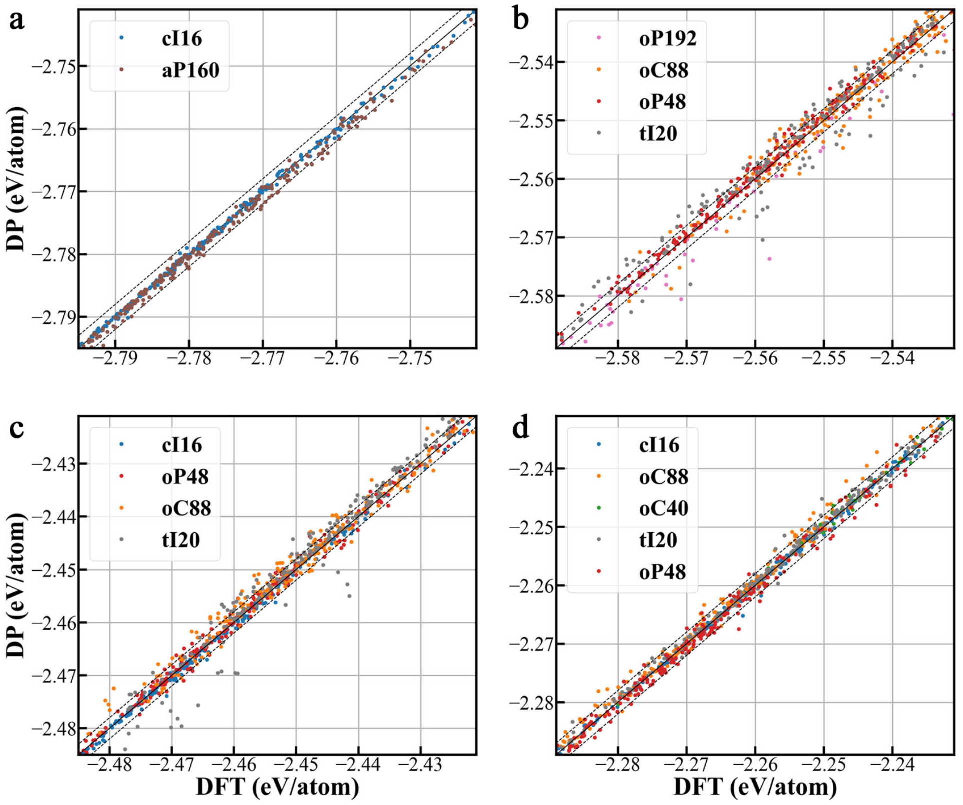

Supplementary Fig. S4. **DP-predicted energy versus DFT-calculated energy of Li structures in the independent testing dataset.** The dash-line indicates the ±2 meV/atom error. **a** 45GPa, **b** 55GPa, **c** 60GPa, **d** 70GPa.

S III. ADDITIONAL RESULTS

A. aP160 and similar structures

During the structure searches, several geometrically similar structures as the aP160 were found. These structures include $P2_1/c$ (8 atoms/cell), $P2_1$ (8 atoms/cell), $C2/m$ (8 atoms/cell), $Cmca$ (16 atoms/cell), and Pc (80 atoms/cell) structures. The $C2/m$ structure was proposed in a recent computational study [20]. These structures possess smaller unit cells and higher symmetry than the aP160. They are different from the aP160 in the arrangement manners of the prisms or the magnitude of distortion of atomic chains. The $C2/m$ and aP160 structures are shown in Supplementary Fig. **S5**(a) and (b), respectively, and all structures are provided in separate files in the POSCAR format.

The enthalpies of these aP160-like structures were calculated using DFT at a high level of accuracy and plotted as a function of pressure relative to the experimental cI16 in Supplementary Fig. **S5**(c). These structures show nearly degenerated enthalpies to that of the aP160 at 50 and 55 GPa, but become enthalpically less favorable at higher or lower pressure.

We have also calculated the free energy for the recently proposed $C2/m$ structure [20] at 45 GPa as shown in Supplementary Fig. **S5**(d). Our results show that the aP160 and the $C2/m$ structures are almost energetically degenerate at 0-300 K and both are less favorable in free energy than the cI16. This contradicts the theoretical results reported in Ref. [20], where the $C2/m$ structure is found to be more stable than the cI16 (by less than 1 meV/atom) at temperatures above ∼200 K. This discrepancy can be attributed to the different choices of parameters in the DFT calculations. Here we adopt more tight parameters to ensure better numerical convergence (see Supplementary Sec. I for computational details for DFT calculations).

An interesting phenomenon observed in the aP160 structure is that the atomic chains are gradually distorted with increasing pressure, similar to that observed during the melting of a material (Supplementary Fig. **S6**). This phenomenon is responsible for the favorable enthalpy of aP160 and is not observed in other aP160-like structures due to the constraints of symmetry as well as the small unit cells. The other phenomenon observed in aP160, as well as other structures described below, is the wide distribution of distances of the nearest-neighbor contacts (Supplementary Fig. **S6**). At 50 GPa, the Li...Li distances are between 2.01 - 2.47 Å. This bond alternation is also found in other structures of Li (e.g. the experimental oC40 and oC88 structures) and is a common feature of Li at high pressure as a result of Peierls distortion [21–23].

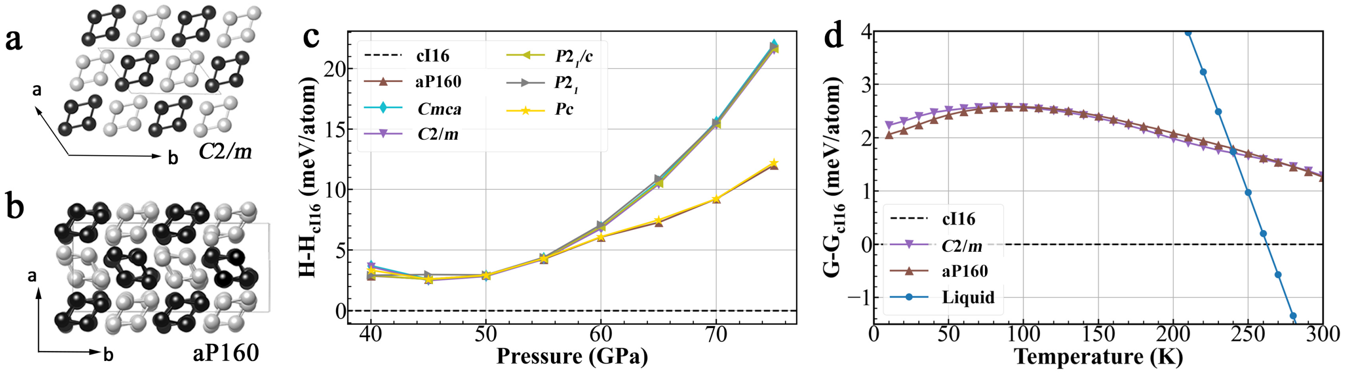

Supplementary Fig. S5. **Structures and relative stabilities of aP160 and similar structures.** **a** $C2/m$ and **b** aP160 structures of Li viewed along the c axis, with the atoms in different rhombic prisms shown in black and grey, respectively. **c** DFT-calculated relative enthalpies per atom of the aP160 and similar structures as a function of pressure between 40 and 75 GPa relative to the cI16 phase. **d** The Gibbs free energies of the $C2/m$, aP160 and liquid phases relative to the cI16 phase versus temperature at 45 GPa using thermodynamic integration (TI) through Deep Potential molecular dynamics (DPMD).

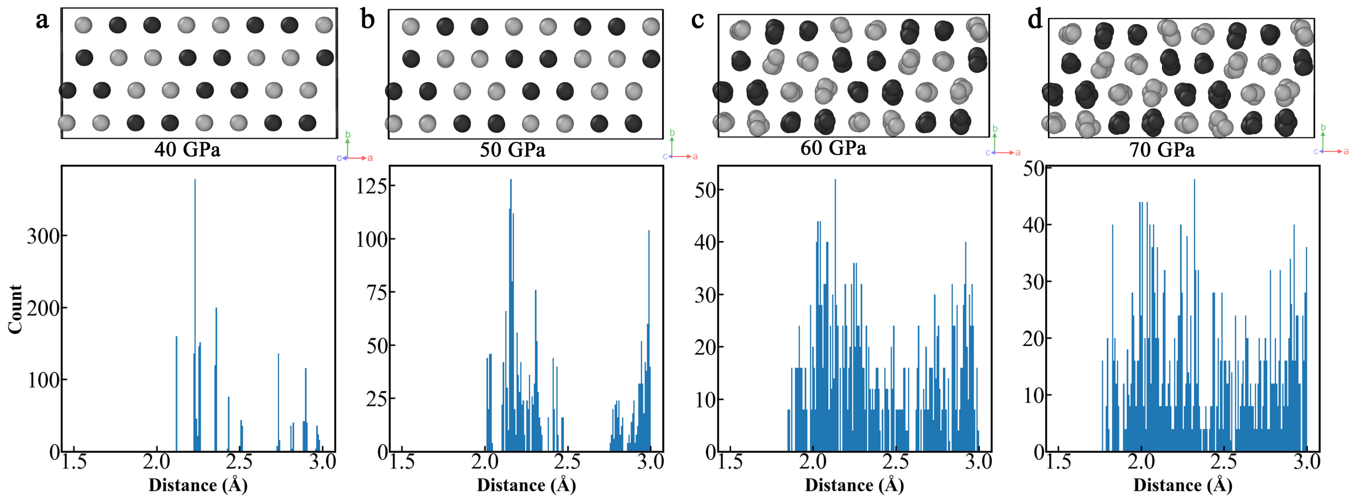

Supplementary Fig. S6. **Structural properties of the aP160.** Structures (upper panel) and histograms of interatomic distances (lower panel) of the aP160 at **a** 40 GPa, **b** 50 GPa, **c** 60 GPa and **d** 70 GPa.

B. Dynamic stabilities of the aP160 and oP192 structures

For the aP160 and oP192, the large unit cell makes DFT-based phonon calculation prohibitively expensive, thus the dynamic stability was examined by DPMD simulations using the NPT ensemble with T = 100 K, and P = 45 and 55 GPa, respectively. The $2 \times 2 \times 2$ supercells (1280 atoms for aP160 and 1536 atoms for oP192) were used in DPMD simulations. Supplementary Fig. **S7** shows the mean square displacement (MSD) and the MD trajectories during the MD simulations for the two structures. For both structures, the MSDs are observed to be almost constant during the 1 ns simulations, which proves that the systems are dynamically stable in the solids with all atoms near their equilibrium positions.

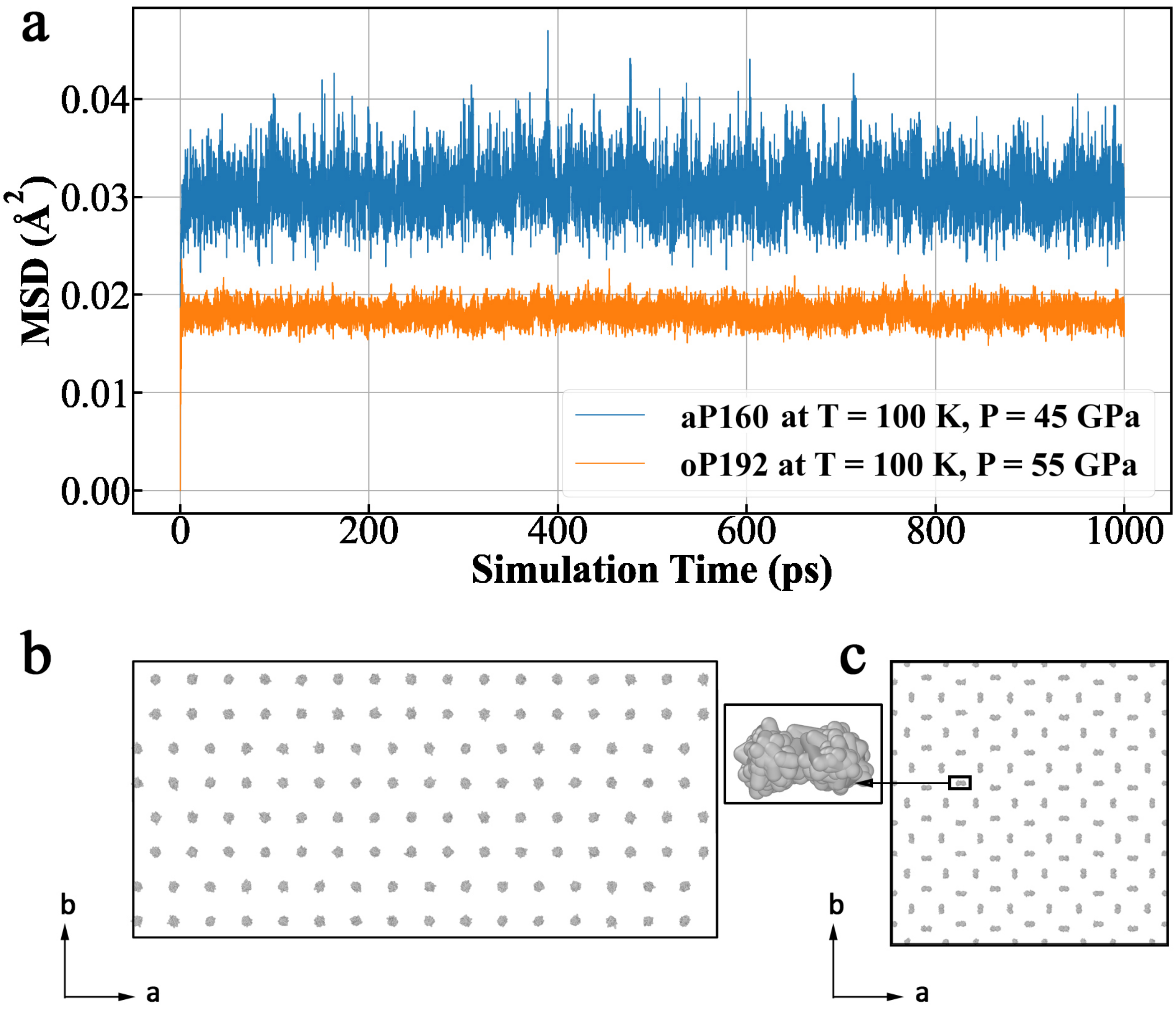

Supplementary Fig. S7. **Dynamic stabilities of the aP160 and oP192 structures. a** The mean square displacement (MSD) during the NPT Deep Potential molecular dynamics (DPMD) simulations of aP160 at T = 100 K, P = 45 GPa and oP192 at T = 100 K, P = 55 GPa. **b** The trajectory snapshots of $2{\times}2{\times}2$ supercell of aP160. (c) The trajectory snapshots of $2{\times}2{\times}2$ supercell of oP192. The atom trajectories are shown by grey lines.

S IV. FREE ENERGY CALCULATIONS

The calculation of Gibbs free energy of a Li phase takes a two-step process:

1. Calculating the free energy of a given Li phase relative to a reference system, whose free energy is known. It is realized by the Hamiltonian thermodynamic integration (HTI) at a reference thermodynamic condition, (T_0, P_0), where the dynamic stability of the Li phase should be guaranteed.

2. Calculating the free energy difference of the Li phase between the target thermodynamic condition (T_1, P_1) and the reference condition (T_0, P_0). It is realized by the thermodynamic integration (TI) along an isobar ($P_0 = P_1$) in this work.

The principles of HTI and TI are introduced in Ref. [18]. The HTI and TI are implemented with the workflow in Deep Potential thermodynamic integration (DPTI) code, which automatically generates the scripts for DPMD simulations conducted by LAMMPS [13], and manages the pre-processes, post-processes and numeric integrations. For all DPMD simulations, the timestep was set to 1 fs. Before conducting HTI, a NPT DPMD simulation was conducted to obtain the proper simulation box at (P_0, T_0). Then a NVT DPMD simulation is conducted at T_0 to check whether the obtained simulation box is reasonable. The NPT DPMD simulation runs up to 1 ns, and the NVT DPMD simulation runs up to 200 ps.

A. Hamiltonian thermodynamic integration

For a solid phase, the reference system is the Einstein crystal [24] with the spring constant $k = 0.045$ eV/Å^2 In the Einstein crystal, atoms are non-interacting. Two intermediate systems are introduced to make the integration path as smooth as possible.

The first intermediate system is the modified Einstein crystal which includes a soft-core Lennard-Jones(LJ) interaction[25]. The potential energy U_{SC} is given by

$$U_{SC}(r) = \eta^n 4\epsilon \left\{ \frac{1}{[\alpha(1-\eta) + (r/\sigma)^6]^2} + \frac{1}{\alpha(1-\eta)^2 + (r/\sigma)^6} \right\}, \tag{4}$$

which repels atoms at short distances. We set $\eta = 0.5$, $\sigma = 1.95$, $\epsilon = 0.3$, $\alpha = 0.5$, and $n = 1.0$ in Eq. (4). The integration path between the Einstein crystal and the first intermediate system, $\lambda \in [0, 1]$, is discretized by $\Delta\lambda = 0.0125$ for $\lambda \in [0.0, 0.1)$, $\Delta\lambda = 0.025$ for $\lambda \in [0.1, 0.2)$, and $\Delta\lambda = 0.2$ for $\lambda \in [0.2, 1.0]$.

The second intermediate system switches on the DP model on top of the first intermediate system. The path between the first and second intermediate systems is discretized by $\Delta\lambda = 0.01$ for $\lambda \in [0.0, 0.05)$, $\Delta\lambda = 0.02$ for $\lambda \in [0.05, 0.15)$, $\Delta\lambda = 0.04$ for $\lambda \in [0.15, 0.35)$, and $\Delta\lambda = 0.065$ for $\lambda \in [0.35, 1.0]$.

The target system is a Li solid phase modeled by DP. The path from the second intermediate system to the target system is discretized by $\Delta\lambda = 0.125$ for $\lambda \in [0.0, 0.75)$, $\Delta\lambda = 0.05$ for $\lambda \in [0.75, 0.9)$, $\Delta\lambda = 0.02$ for $\lambda \in [0.90, 0.96)$, and $\Delta\lambda = 0.01$ for $\lambda \in [0.96, 1.0]$.

MD simulations at each λ run up to 300 ps. The HTI for solid phases was conducted at $T_0 = 150$ K, except that for oC88 and oP192 structure at 55 GPa, $T_0 = 50$ K. The numbers of atoms used in the HTI of cI16, oC88, aP160, tI20, oP48, and oP192 structures are 432, 352, 320, 320, 384, and 768, respectively.

For the HTI of Li liquid phase, the reference system is the ideal gas. The constructions of the two intermediate systems are similar to those in the HTI for solid phases. The first intermediate system switches on the soft-core LJ potential on top of the ideal gas, and the second system additionally switches on DP. The parameters in the soft-core potential in Eq.(4) are set to $\eta = 0.5$, $\sigma = 1.95$, $\epsilon = 0.01$, $\alpha = 0.5$, and $n = 1.0$.

The integration path between the ideal gas and the first intermediate system is discretized by $\Delta\lambda = 0.003$ for $\lambda \in [0.00, 0.03)$, $\Delta\lambda = 0.005$ for $\lambda \in [0.03, 0.06)$, $\Delta\lambda = 0.01$ for $\lambda \in [0.06, 0.16)$, $\Delta\lambda = 0.02$ for $\lambda \in [0.16, 0.30)$, and $\Delta\lambda = 0.05$ for $\lambda \in [0.30, 1.00)$. The path between the first and second intermediate systems is discretized by $\Delta\lambda = 0.002$ for $\lambda \in [0.00, 0.006)$, $\Delta\lambda = 0.004$ for $\lambda \in [0.006, 0.03)$, $\Delta\lambda = 0.01$ for $\lambda \in [0.03, 0.16)$, $\Delta\lambda = 0.03$ for $\lambda \in [0.16, 0.40)$, and $\Delta\lambda = 0.06$ for $\lambda \in [0.40, 1.00)$. The path from the second intermediate system to the target system is discretized by $\Delta\lambda = 0.125$ for $\lambda \in [0.00, 0.75)$, $\Delta\lambda = 0.05$ for $\lambda \in [0.75, 0.90)$, and $\Delta\lambda = 0.01$ for $\lambda \in [0.90, 1.00)$.

During the HTI of the liquid phase, The number of atoms used in the liquid phase HTI is 432. MD simulations at each λ run up to 600 ps. The starting temperature T_0 for the liquid phase is set to 400 K.

B. Thermodynamic integration

After obtaining the relative Gibbs free energy of a phase at the (T_0, P_0), we use TI to calculate the Gibbs free energy of the phase in a range of T_1 along the isobar. MD simulations in TI run up to 500 ps. For solid phases, the range of T_1 is [10, 300] K. For the liquid phase, the range of T_1 is [200, 400] K. The interval for T_1 is 10 K for TI of all phases.

[1] G. Kresse and J. Furthmüller, Phys. Rev. B **54**, 11169 (1996).
[2] P. E. Blöchl, Phys. Rev. B **50**, 17953 (1994).
[3] J. P. Perdew and Y. Wang, Phys. Rev. B **45**, 13244 (1992).
[4] J. P. Perdew, K. Burke, and M. Ernzerhof, Phys. Rev. Lett. **77**, 3865 (1996).
[5] A. Togo and I. Tanaka, Script. Mater. **108**, 1 (2015).
[6] X. Gonze, B. Amadon, P.-M. Anglade, J.-M. Beuken, F. Bottin, P. Boulanger, F. Bruneval, D. Caliste, R. Caracas, M. Côté, T. Deutsch, L. Genovese, P. Ghosez, M. Giantomassi, S. Goedecker, D. Hamann, P. Hermet, F. Jollet, G. Jomard, S. Leroux, M. Mancini, S. Mazevet, M. Oliveira, G. Onida, Y. Pouillon, T. Rangel, G.-M. Rignanese, D. Sangalli, R. Shaltaf, M. Torrent, M. Verstraete, G. Zerah, and J. Zwanziger, Comput. Phys. Commun. **180**, 2582 (2009).
[7] C. Hartwigsen, S. Goedecker, and J. Hutter, Phys. Rev. B **58**, 3641 (1998).
[8] Y. Zhang, H. Wang, W. Chen, J. Zeng, L. Zhang, H. Wang, and W. E, Comput. Phys. Commun. **253**, 107206 (2020).
[9] X. Wang, Y. Wang, L. Zhang, F. Dai, and H. Wang, Nucl. Fusion **62**, 126013 (2022).
[10] D. Kingma and J. Ba, in *Proceedings of the International Conference on Learning Representations (ICLR)* 1 (2015).
[11] M. Abadi, A. Agarwal, P. Barham, E. Brevdo, Z. Chen, C. Citro, G. S. Corrado, A. Davis, J. Dean, M. Devin, *et al.*, arXiv: Distributed, Parallel, and Cluster Computing. arXiv:1603.04467 (2015).
[12] J. Han, L. Zhang, R. Car, and W. E, Commun. Comput. Phys. **23**, 629 (2018).
[13] A. P. Thompson, H. M. Aktulga, R. Berger, D. S. Bolintineanu, W. M. Brown, P. S. Crozier, P. J. in 't Veld, A. Kohlmeyer, S. G. Moore, T. D. Nguyen, R. Shan, M. J. Stevens, J. Tranchida, C. Trott, and S. J. Plimpton, Comput. Phys. Commun. **271**, 108171 (2022).
[14] H. Wang, L. Zhang, J. Han, and W. E, Comput. Phys. Commun. **228**, 178 (2018).
[15] L. Zhang, J. Han, H. Wang, R. Car, and E. Weinan, Phys. Rev. Lett. **120**, 143001 (2018).
[16] C. L. Guillaume, E. Gregoryanz, O. Degtyareva, M. I. McMahon, M. Hanfland, S. Evans, M. Guthrie, S. V. Sinogeikin, and H. Mao, Nat. Phys. **7**, 211 (2011).
[17] M. Frost, J. B. Kim, E. E. McBride, J. R. Peterson, J. S. Smith, P. Sun, and S. H. Glenzer, Phys. Rev. Lett. **123**, 065701 (2019).
[18] C. Vega, E. Sanz, J. Abascal, and E. Noya, J. Phys. Condens. Matter. **20**, 153101 (2008).
[19] L. Zhang, H. Wang, R. Car, and E. Weinan, Phys. Rev. Lett. **126**, 236001 (2021).
[20] Y. Wang, J. Wang, A. Hermann, S. Pan, J. Shi, H.-T. Wang, D. Xing, and J. Sun, Phys. Rev. B **105**, 214101 (2022).
[21] J. Lv, Y. Wang, L. Zhu, and Y. Ma, Phys. Rev. Lett. **106**, 015503 (2011).
[22] Y. Yao, J. S. Tse, and D. D. Klug, Phys. Rev. Lett. **102**, 115503 (2009).
[23] J. B. Neaton and N. W. Ashcroft, Nature **400**, 141 (1999).
[24] D. Frenkel and A. J. Ladd, J. Chem. Phys. **81**, 3188 (1984).
[25] T. Steinbrecher, I. Joung, and D. A. Case, J. Comput. Chem. **32**, 3253 (2011).
