# Supplementary material for: Data-driven prediction of complex crystal structures of dense lithium
Source: Nat Commun. 2023 May 22;14:2924. doi: 10.1038/s41467-023-38650-y (PMC10203143; doi:10.1038/s41467-023-38650-y)
Supplement: Supplementary file 3 — Description of Additional Supplementary Files [file 41467_2023_38650_MOESM3_ESM.pdf]

Description of Additional Supplementary Files

File Name: Supplementary Data 1

Description: Crystal structure files of lithium in the POSCAR format.
